# Structural analysis and treatment of acute liver injury of polysaccharides from the *Radix Actinidiae Chinensis*

**DOI:** 10.3389/fphar.2025.1544302

**Published:** 2025-02-24

**Authors:** Zhongpeng Ding, Qianyi Ying, Jing Lei, Ningchen Zhang, Yu Xia, Yilin Du, Beihua Xu, Senlin Shi

**Affiliations:** ^1^ College of Pharmaceutical Sciences, Zhejiang Chinese Medical University, Hangzhou, China; ^2^ Pharmacy Department, Lishui Second People’s Hospital, Lishui, China

**Keywords:** artificial neural network, *Radix Actinidiae Chinensis*, polysaccharide, acute liver injury, process optimization

## Abstract

**Objective:**

To investigate the therapeutic effects of the PRAC on acute liver injury and its potential as an ingredient in drugs and nutraceuticals.

**Methods:**

Microwave-assisted extraction technology combined with Box-Behnken model combined with the three kinds of artificial neural networks was used to optimize PRAC extraction process. Characterize the structure and composition of PRAC. In order to prevent PRAC from being degraded by the gastrointestinal environment, PRAC-loaded liposomes were fabricated. The efficacy of PRAC-loaded liposomes was evaluated by three acute liver injury animal models prepared according to different mechanisms.

**Results:**

The best yield of PRAC was 4.49%, and the purity reached up to 86.53% by optimizing the microwave parameters using Box-Behnken model combined with the three kinds of artificial neural networks. PRAC was characterized as a galactan having a backbone consisting predominately of →4)-D-Gal*p*-(1→ and →4)-D-Glc*p*-(1→ with a molecular weight of 12.713 kDa. The PRAC-loaded liposome obtained had a size about 340 nm with a polydispersity index 0.234. The entrapment efficiency was 70.12% and the drug loading was 1.24%. Liposomes can be fully released in the gastrointestinal environment within 12 h and have long-term stability at 4°C. The therapeutic effect of PRAC liposomes on acute liver injury was confirmed by three animal models of acute liver injury.

**Conclusion:**

PRAC is a potential drug for the treatment of acute liver injury.

## 1 Introduction

The RAC is the dried rhizome of actinidia chinensis planch, which has been used as an ingredient in food and drug for more than 1,000 years (He X. R. et al., 2019). Current research suggests that the PRAC are the main active components RAC, which have anti-tumor, antioxidant, anti-aging and immunomodulatory effects, etc. (Zhang L. et al., 2023). Most of the current studies on PRAC have focused on antitumor effects, but there are only few studies on its therapeutic effects on acute liver injury caused by oxidative stress, lipid metabolism, inflammatory response, and immune regulation (Fang et al., 2020; Qiu K. J. et al., 2023; Xu et al., 2009; Zhu et al., 2013).

Liver injury is an attack on the liver by viruses, bacteria, drugs, alcohol, and other pathogenic factors, resulting in impaired liver function, causing a series of symptoms, and even death (Hosack et al., 2023; Loomba et al., 2021; Yang et al., 2022). Due to the complex hepatic pathophysiology and the different progression levels of these conditions, developing a drug for their treatment remains a major challenge for modern pharmacology (Parlati et al., 2021). On the other hand, numerous studies have proven the benefits that specific polysaccharides can provide as an alternative to prevent and/or reverse acute liver injury, with reduced adverse effects (Riaz et al., 2023; Zhong et al., 2022). However, due to factors such as low solubility and stability, low absorption, the medicinal potential of polysaccharides is highly affected, which consequently limits their therapeutic efficacy. Nowadays, advances in nanomedicine have led to the development of improved ways of dosing polysaccharides by using nanocarriers as a transport vehicle to enhance the absorption and bioavailability of these active compounds in the hepatic environment to carry out their therapeutic function. These nanomedicines allow for increased intestinal absorption, increased bioavailability of the active ingredient in the liver and limited side effects due to *in situ* accumulation of the drug (Heya et al., 2023).

The first challenge in polysaccharide research lies in the complex process of polysaccharides extraction, because they coexist with other biological substances in organisms (Huang et al., 2021). It is difficult to evaluate the influence of different extraction process parameters on the concentration of active components in the extraction process (Leong et al., 2021). The Box-Behnken model is often used in extraction process optimization to find the best process parameters, which essentially involves determining the coefficients of linear polynomials by designing experiments. However, the polynomial only reaches the quadratic term, which limits its ability to describe the relationship between some complex independent variables and the response variables (Qiu J. J. et al., 2023; Qiu J. J. et al., 2024). BP artificial neural network was proposed by Rumelhart and McCelland in 1986 as a multilayer feedforward network trained by an error backpropagation algorithm (Zhang J. H. et al., 2023), and it is now one of the most widely used artificial neural network models. Unfortunately, it has been find that people this approach often leads to the final result in local minimum value, with some problems such as low learning efficiency and sensitive parameters (Rathore et al., 2023). GA is an optimization search algorithm based on natural selection and genetic principles, which introduces “survival of the fittest” into the parameter optimization process (Zhang et al., 2020). The algorithm tends to encode the chromosomes of the population, evolve generation by generation, produce the optimal individuals, and finally decode the optimal individuals to obtain the optimal solution. GA starts at multiple points and is computed instead of being restricted to one, thus effectively preventing it from getting trapped in a local optimal solution. Therefore, we can use the global search performance of GA to find the region of the optimal solution to the problem, and then use the error backpropagation method to find the optimal solution (Badhwar et al., 2020; Duvvuri and Anmala, 2019; Irani and Nasimi, 2011). ACO is a method that simulates the transmission of pheromone among ants to optimize target path search. Subsequently, the prediction performance of the neural network can be further improved by optimizing the pheromone by GA, followed by optimizing the weights and thresholds of the BP neural network by ACO (Ong et al., 2019; Yuan et al., 2019).

In this study, the Box-Behnken design and three artificial neural network models were used to optimize the PRAC extraction process to improve the yield. The composition of PRAC was elucidated by isolation, purification and structural analysis. To fully utilize the pharmacological potential of PRAC, PRAC-loaded liposomes were prepared. Finally, the hepatoprotective effect of PRAC was demonstrated in three animal models of acute liver injury. Our findings may provide an important basis for the application of PRAC and the development of potential therapeutic strategies for acute liver injury.

## 2 Materials and methods

### 2.1 Materials

*Radix Actinidiae Chinensis* was purchased from Beijing Tongrentang Co., Ltd. (Cat. No. 20230903, Produced in Bozhou, Anhui, China); Polyamides and D900 resins were purchased from MeilunBio Co., Ltd (Cat. No.412A0101); Coomassie Brilliant Blue, M-hydroxydiphenyl, and Congo Red were bought from Shanghai Yuanye Biotechnology Co., Ltd (Cat. No. S25IS226890, D19HS204503, O11IS228299); Lecithin was acquired from Shanghai Advanced Vehicle Technology Co., Ltd (Cat. No.20230810); Cholesterol was purchased from Shanghai Siji Biological Products Co., Ltd (Cat. No. 20230726); PEG2000 was purchased from Sinopharm Group Chemical Reagent Co., Ltd (Cat. No. 20231018); D-galactosamine and concanavalin were purchased from Beijing Biodee Biotechnology Co., LTD. (Cat. No. 412A0101); Alanine aminotransferase test kits, aspartate aminotransferase test kits, Malondialdehyde detection kit, Superoxide dismutase detection kit, Tumor necrosis factor detection kits and Gamma interferon detection kits were bought from Shanghai Yuanye Biotechnology Co., Ltd (Cat. No. MT-0190R1, MT-0577R1, MT-0532R1, MT-0543R1).

### 2.2 Animals and ethics statement

ICR mice (half male and half female, 18 ± 2 g, 6–8 weeks) were provided by the Laboratory Animal Center of Zhejiang Chinese Medical University (No. SCXK (Zhe)2022-0005). Experimental animals were kept under constant ambient conditions (room temperature 21°C ± 1°C, relative humidity 40%–70%, light-dark cycle 12 h). All mice had access to food and water. All animal experiments were performed according to protocols approved by the Laboratory Animal Center of Zhejiang Chinese Medical University (No. 20240304-12).

### 2.3 Comparison of four extraction methods and polysaccharide content detection

Reflux extraction, ultrasound-assisted extraction, cold soak extraction, and microwave-assisted extraction are commonly used extraction methods. In this experiment, the effects of these four extraction methods on the yield of PRAC were compared.

Cold soaked extraction: The PRAC yield was calculated after accurately weighing W_1_ of RAC powder (65 mesh), adding 30% ethanol at the ratio of 1:10 w/v, and soaking at room temperature for 24 h.

Reflux extraction: W_1_ of RAC powder (65 mesh) was precisely weighed, 10 times of 30% ethanol was added, and the mixture was refluxed for 60 min in a round-bottom flask at 80°C (Hu et al., 2018).

Ultrasonic-assisted extraction: The W_1_ of RAC powder (65 mesh) was mixed with 10 times of 30% ethanol, and placing the flask in a bath sonicator for 60 min. The sonication parameters were set as follows: the power was 300 W, and the temperature was 80°C (Chen et al., 2020).

Microwave extraction: For this experiment, W_1_ of RAC powder was accurately weighed, placed in a round-bottom flask with the 30% ethanol at the ratio of 1:10 w/and then microwaved for 60 min at 80°C at a power of 300 W (Bagade and Patil, 2021).

The PRAC were recovered by centrifugating the above filtrates at 8000 g for 15 min to collect the supernatants, followed by precipitating them by adding 95% ethanol to the supernatant to a final ethanol concentration of 75%. Then the precipitated PRAC were harvested by centrifugation at 8000 g for 15 min, and then dried at 60°C under vacuum to a constant weight (W_2_). The extraction yield was determined using Equation 1.

$$yield\left(\%\right)=W_{2}/W_{1}\times 100\%$$ (1)

The polysaccharide content was measured by a phenol-sulfuric acid method using glucose as a standard. The purity of PRAC (W_3_) was determined using Equation 2.

$$purity\left(\%\right)=W_{3}{/W}_{2}\times 100\%$$ (2)

### 2.4 Single-factor experimental design

A single factor experimental design was used in which only one variable was changed at a time to determine the effective ranges for the optimization experiment. The effect of each factor in the microwave-assisted extraction process on PRAC yields and purities were investigated in the following order: liquid-solid ratio (10:1, 14:1, 18:1, 20:1), temperature (40°C, 60°C, 80°C, 100°C), time (30 min, 60 min, 90 min, 120 min) and microwave power (100 W, 200 W, 300 W, 400 W). The sugar content and purity of PRAC were quantified by the analytical procedures described in Section 2.3.

### 2.5 Box-Behnken experimental design

Preliminary ranges of liquid-to-solid ratio, microwave power, extraction temperature, and extraction time were determined based on single-factor experiments. In order to obtain the best combination of the four variables, a Box-Behnken model with four factors and three levels was designed using the Design-Expert 12 software (Table 1). Using the PRAC yield as the response value, the regression model was optimized by interaction analysis of the response surface factors, and the final extraction parameters of PRAC were obtained.

**TABLE 1:** True values from 29 extraction experiments with predicted values from 4 models.

Run	Liquid-to-solid ratio (mL/g)	Ultrasonic power (W)	Extraction temperature (°C)	Processing time (min)	Yield (%) Actual	Box-Behnken predicted	BP Predicted	GA-BP Predicted	GA-ACO-BP predicted
1	18	80	90	400	2.945	3.010	3.249	3.006	2.995
2	18	60	60	400	2.831	2.670	2.976	2.702	2.831
3	18	100	90	500	3.907	3.820	3.865	3.723	3.895
4	18	80	90	400	2.921	3.010	3.249	3.006	2.995
5	22	80	60	400	3.060	3.300	3.078	3.121	3.060
6	18	80	60	300	2.962	2.840	3.146	2.892	2.962
7	22	80	120	400	3.678	3.480	3.695	3.518	3.678
8	14	100	90	400	3.353	3.220	3.583	3.335	3.371
9	14	80	120	400	3.083	2.970	3.119	3.030	3.249
10	14	80	60	400	2.305	2.620	3.276	2.282	2.305
11	18	80	60	500	3.472	3.330	2.990	3.531	3.383
12	18	80	90	400	2.914	3.010	3.249	3.006	2.995
13	18	100	60	400	3.729	3.590	3.838	3.659	3.729
14	14	80	90	300	2.612	2.480	3.123	2.605	2.240
15	18	80	90	400	3.116	3.010	3.249	3.006	2.995
16	18	60	90	500	2.802	2.720	3.025	2.821	2.802
17	18	100	120	400	4.170	4.150	3.755	4.173	4.170
18	18	100	90	300	3.485	3.700	3.581	3.448	3.485
19	22	80	90	300	3.619	3.250	3.470	3.055	3.619
20	22	100	90	400	3.870	4.050	3.657	3.570	3.870
21	18	60	120	400	2.683	2.640	3.107	2.728	2.683
22	18	80	90	400	3.151	3.010	3.249	3.006	2.995
23	22	80	90	500	3.355	3.310	3.210	3.219	3.027
24	22	60	90	400	2.412	2.600	3.097	2.323	2.412
25	14	60	90	400	2.353	2.230	3.097	2.196	2.394
26	18	60	90	300	2.149	2.370	3.096	2.158	2.245
27	18	80	120	500	3.160	3.340	3.494	3.262	3.223
28	14	80	90	500	2.701	2.890	3.224	2.905	2.705
29	18	80	120	300	3.167	3.360	3.153	3.320	3.328

### 2.6 Comparation of the three kinds of artificial neural networks

MATLAB R2020b software was used to construct three different artificial neural network models. The input layer was composed of four neurons: liquid-to-solid ratio, ultrasound power, extraction temperature and extraction time, and the output layer was composed of one neuron: polysaccharide yield. The experimental data were divided into a training set (21 datasets) and a test set (8 datasets). To estimate the number of neurons in the hidden layer, we utilize Equation 3.

$$Hidden\;num=sqrt\left(m+n\right)+a$$ (3)

Where m is the number of neurons in the input layer, n is the number of neurons in the output layer, and a = 1 to 10.

Three different artificial neural networks were trained, fitted, and the best process conditions were found. The performance of the model was evaluated by the R^2^, MAE, and RMSE, which were calculated using Equations 4–6.

$$R^{2}=ESS/TSS=1-RSS/TSS=1-{\sum_{i=1}^{n}\left(Y_{i,a}-{\tilde{Y}}_{i,p}\right)}^{2}/{\sum_{i=1}^{n}\left({\bar{Y}}_{a}-{\tilde{Y}}_{i,p}\right)}^{2},k=1,2$$ (4)

$$MAE=\left(1/n\right)\sum_{i=1}^{n}\left|Y_{i,a}-{\tilde{Y}}_{i,p}\right|,k=1,2$$ (5)

$$RMSE=\sqrt{\left(1/n\right)\sum_{i=1}^{n}{\left(Y_{i,a}-{\tilde{Y}}_{i,p}\right)}^{2}},k=1,2$$ (6)

Among them, the ${\tilde{Y}}_{i,p}$ is each model forecast, $Y_{i,a}$ is the actual value, ${\bar{Y}}_{a}$ is the average of the data set. RSS, ESS, and TSS represent the sum of regression squares, residual squares, and total squares, respectively.

### 2.7 Decolorization and purification of PRAC

Because of the dark color of PRAC and its aqueous solution, it is necessary to decolorize the polysaccharide. The pH of 2 mg/mL PRAC solution was adjusted to 5, adding polyamide until the ratio was PRAC: polyamide = 60 mg: 1 g, and the mixture was shaken at room temperature for 80 min (100 r/min) for decolorization.

The polysaccharides were purified by the anion exchange resin column (4.0 cm × 2.0 cm), with a PRAC concentration of 8 mg/mL and a loading volume of 25 mL. The pH of polysaccharide solution was adjusted to 8 with 1% NaOH solution, and the sample was loading at a flow rate of 0.4 mL/min, and the column temperature was controlled to 33°C. Then 60 mL of 0.6 mol/L NaCl solution was eluted at a flow rate of 0.8 mL/min. The eluent was collected, concentrated and precipitated with ethanol to purify the PRAC.

### 2.8 Characterization of PRAC

#### 2.8.1 Scanning electron microscopy

A small amount of dried PRAC was adhere to the metal by cellophane tape, and sputtered with a thin layer of gold using a gold sputter, and then placed under a SEM to observe the morphology of PRAC.

#### 2.8.2 Monosaccharide composition

The PRAC sample was mixed with 2 mL TFA solution (2 mol/L) in test tube and hydrolyzed at 105°C for 6 h. The tube was cooled to room temperature. After centrifugation at 4,000 g for 5 min, the supernatant was collected and blow-dried under nitrogen, and then washed with methanol under nitrogen for three times to remove the excessive TFA. Then 5 mL of ultrapure water was added in the dry powder for derivatization. In order to, 0.20 ml of the above sample solution was mixed with 0.20 mL of PMP methanol solution (0.5 mol/L) and 0.20 mL of NaOH solution (0.3 mol/L), followed by heating at 70°C for 60 min. And then 0.20 mL HCl solution (0.3 mol/L) was added to neutralize the residual base when the mixture was cooled to room temperature. Subsequently, the above solution was mixed with 1.0 mL chloroform for 30 s, centrifuged at 2000 g for 5 min to remove the chloroform layer, and repeated for three times. The supernatant was finally diluted to 5 mL with ultrapure water for HPLC analysis.

The types and contents of monosaccharides were determined by HPLC (Agilent 1,200). The mobile phase consisted of acetonitrile (A) and 0.05 mol/mL phosphate buffer (pH = 6.8, B). The gradient elution was set as follows: 0–15 min: 14% to 16%A, 15–26 min: 16%-24%A, 26–35 min: 24%–50%A. Monosaccharides was detected by the chromatographic column of ZORBAX Eclipse XDB-C18 (5 μm, 250 mm × 4.6 mm) at 1 mL/min flow rate and the temperature of 30°C with 20 μL of sample injection volume.

#### 2.8.3 Measurement of the protein and uronic acid contents

The absorbance of PRAC at 200–800 nm was measured using a UV-vis spectrophotometer. The protein content of PRAC was determined by the coomassie brilliant blue method. The content of uronic acid was determined by the m-hydroxydiphenyl method.

#### 2.8.4 Congo red experiment

The PRAC sample (1 mL, 2.5 mg/mL) was mixed with 1 mL congo red reagent (80 μmol/L). A series of solutions were made with final NaOH concentrations ranging from 0 to 0.5 mol/L by adding 1 mol/L NaOH solution to the above solution. Finally, a UV spectrophotometer was used to determine the maximum absorption wavelength in the range of 200–800 nm, using congo red as a control, to determine whether the structure of PRAC contained a three-stranded helix conformation.

#### 2.8.5 FTIR spectroscopy analysis

Approximately 1 mg PRAC was mixed with KBr at the ratio 1:10, followed by grounding into a fine powder and pressing into a thin tablet. Infrared spectrometer was used to measure the transmittance in the range of 4,000–500 cm^-1^, using KBr as background spectra.

#### 2.8.6 X-ray diffraction

The dried PRAC was put into an XRD. The measurement conditions were set as follows: copper target (k = 1.5406 Å), pipe pressure 40 kV, Angle range 5°–80°(2θ), and scanning speed 1°/min.

#### 2.8.7 Molecular weight analysis

The molecular weight and homogeneity of PRAC were determined using a GPC system. A waters Ultrahydrogel 1,000A was selected for the analysis. The concentration of the sample solution was 1.0 mg/mL. The chromatographic conditions were consistent with previously reported methods (Tu et al., 2022). The dextran standards of Mw: 430, Mw: 4,290, Mw: 20,600, Mw: 82,500, Mw: 176,000 and Mw: 330,000 were used to establish standard curves for the relative molecular mass calculation.

#### 2.8.8 Methylation analysis

The PRAC (5 mg) was entirely methylated, hydrolyzed, reduced and acetylated to obtain partially methylated alditol acetates, which were detected by GC–MS equipped with a RXI-5 SIL MS column (60 m × 0.25 mm, 0.25 μm) based on literature (Liang et al., 2024).

#### 2.8.9 Nuclear magnetic resonance

The chemical composition of the polysaccharide was determined using NMR by taking 20 mg of PRAC dissolved in 0.8 mL D_2_O. The analyses were performed using MestReNova 14.0 software.

### 2.9 Preparation of PRAC-loaded liposomes

PRAC-loaded liposomes were prepared by reverse evaporation. Briefly, 7.5 mg of PRAC and 22.5 mg of PEG-2000 were dissolved in 10 mL ultrapure water to prepare an aqueous solution. The 150.0 mg of lecithin and 37.5 mg of cholesterol were dissolved in 40 mL of diethyl ether. The mixture and the aqueous solution were then poured into a beaker and stirred at 12,000 r/min for 2.5 min to make a homogeneous liquid of W/O type. The ether was evaporated by rotary evaporation to obtain a liposome film at 25°C. After complete removal of the organic solvent. 5 mL of ultrapure water was added to the lipid film as a hydration solution, homogenized at high pressure, and then the solution was diluted to 50 mL with ultrapure water to obtain PRAC loaded liposomes.

### 2.10 Characterization of PRAC-loaded liposomes

The diameter and morphology of the PRAC-loaded liposomes were observed by TEM. The liposome solution was dropped onto carbon-coated copper grids, dried naturally in air, and the morphology was visualized by TEM. The particle size, Zeta potential and PDI of the PRAC-loaded liposomes were measured using dynamic light scattering.

### 2.11 Loading capacity and encapsulation efficiency of PRAC-loaded liposomes

To determine the encapsulation efficiency and drug loading, anion exchange resin was used to separate the liposomes and free polysaccharides due to the negatively charged nature of the liposomes. Briefly, 5 mL of the liposome solution was eluted with 1 mol/L NaCl solution, and the free polysaccharide content was determined by the sulfuric acid-phenol method. LC and EE were calculated using Equations 7, 8.

$$EE=\left(W_{2}-W_{1}\right)/W_{2}\times 100\%$$ (7)

$$LC=\left(W_{2}-W_{1}\right)/\left(W_{3}+W_{2}\right)\times 100\%$$ (8)

Where W_2_ is the total polysaccharide mass, W_1_ is the free polysaccharide mass, and W_3_ is the carrier mass.

### 2.12 *In vitro* release of PRAC-loaded liposomes

The dialysis bag containing 5 mL of the liposome solution was placed into 50 mL of PBS solution at pH = 2 and pH = 7.6 (to simulate the human stomach and small intestine environment), and incubated at 37°C with slow agitation. Samples were collected at 0 h, 1 h, 2 h, 4 h, 8 h, 12 h, 24 h, 36 h, and 48 h. The cumulative rate of polysaccharide release was determined using the phenol-sulfuric acid method.

### 2.13 The preliminary stability of PRAC-loaded liposomes

An equal volume of ultrapure water was added to the liposome solution and centrifuged at 3,000 rpm for 30 min. The UV spectra of liposomes were recorded before and after dilution. *Ke* was calculated through Equation 9.

$$Ke=\left(A_{0}-A\right)/A_{0}$$ (9)

Where A_0_ is the absorbance before centrifugation and A is the absorbance after centrifugation.

The thermal stability of PRAC-loaded liposomes was investigated in terms of particle size, encapsulation efficiency and *Ke* at 37°C, 45°C, 60°C and autoclave sterilization. According to Chinese Pharmacopoeia 2020 edition, artificial gastric fluid and artificial intestinal fluid were prepared respectively. The liposomes were diluted with ultrapure water, PBS buffer (pH = 7.4), artificial gastric fluid and artificial intestinal fluid, respectively. The physical state changes of the liposomes in different media solutions were observed within 24 h. The liposomes were incubated at 4°C and 25°C for 6 months, and the changes in encapsulation efficiency, stability coefficient, and pH were measured to evaluate their long-term stability.

### 2.14 Therapeutic effect of PRAC-loaded liposomes on acute liver injury

#### 2.14.1 Acute liver injury caused by CCl_4_

Seventy-two ICR mice, half male and half female, were randomly divided into 6 groups (*n* = 12): control group, model group, biphenyl diphosphonate group (150 mg/kg), liposome low-dose group (50 mg/kg), liposome medium-dose group (100 mg/kg) and liposome high-dose group (200 mg/kg). The drug was administered orally for 7 days. One hour after the last administration, the mice in the model group and each administration group were intraperitoneally injected with 0.1 mL/kg CCl_4_ solution, and the mice in the control group were injected with the same amount of blank solution. After 24 h, blood samples were collected from the mice, and serum was separated for ALT and AS detection. After blood collection, the animals were executed, and the livers were removed for determination of SOD and MDA levels and for observation of pathological changes in liver tissue.

#### 2.14.2 Acute liver injury caused by D-gal

Animal selection and grouping were the same as in 2.14.1. The drug was administered continuously for 7 days. One hour after the last administration, mice in the model group and each administration group were intraperitoneally injected with 1,000 mg/kg D-gal solution, and mice in the control group were injected with the same amount of blank solution. After 24 h, blood samples were collected from the mice, and serum was separated for ALT and AST detection. After blood collection, the animals were executed, and the liver was removed to observe the pathological changes of liver tissue.

#### 2.14.3 Acute liver injury caused by ConA

Animal selection and grouping were the same as in 2.14.1. On the first day, mice in the model group and each administration group were injected with ConA 20 mg/kg via the tail vein, and then administered 4 h later for 7 consecutive days. On the 8th day, ConA 10 mg/kg was injected into the tail vein of mice in the model group and each administration group. And after 8 h, blood samples were collected, and the serum was separated for the detection of ALT, AST, IFN-γ, and TNF-ɑ. After blood collection, the animals were executed, and the liver was removed, and the pathological changes of the liver histology were observed.

### 2.15 Statistics analysis

Experiments were performed independently in triplicate and the data were represented as mean ± SD using GraphPad Prism (Version 8.0) and the Origin 2022 software for statistical analysis and drawing. Two-group com-parisons were evaluated by unpaired two-tailed Student’s *t* tests, and multiple comparisons were performed by ANOVA. A *p*-value <0.05 was regarded as a statistically difference.

## 3 Results

### 3.1 Comparison of extraction methods

In this study, four methods were used to extract the polysaccharides. The yield of polysaccharide was 1.76% ± 0.02% by reflux extraction, 3.25% ± 0.03% by ultrasound-assisted extraction, 0.82% ± 0.01% by cold-soaked extraction, and 4.10% ± 0.12% by microwave assisted extraction (Tang et al., 2023). Increased temperature contributed to the higher yield of PRAC, and the cold-soaked extraction resulted in a lower yield of PRAC due to the lower extraction temperature. Ultrasound-assisted extraction uses the cavitation effect of ultrasound to destroy the plant structure and release the polysaccharide (Wang et al., 2023). Microwave assisted extraction increases the temperature and pressure inside the plant cells due to microwave radiation, which breaks the cell wall and releases the polysaccharide. Moreover, thanks to the heating procedure during microwave-assisted extraction, the yield of PRAC extracted by microwave-assisted extraction was the highest, so the microwave-assisted extraction method was selected in this experiment (Picot-Allain et al., 2021; Zhang Y. L. et al., 2023).

### 3.2 Single-factor experimental design

**FIGURE 1:**
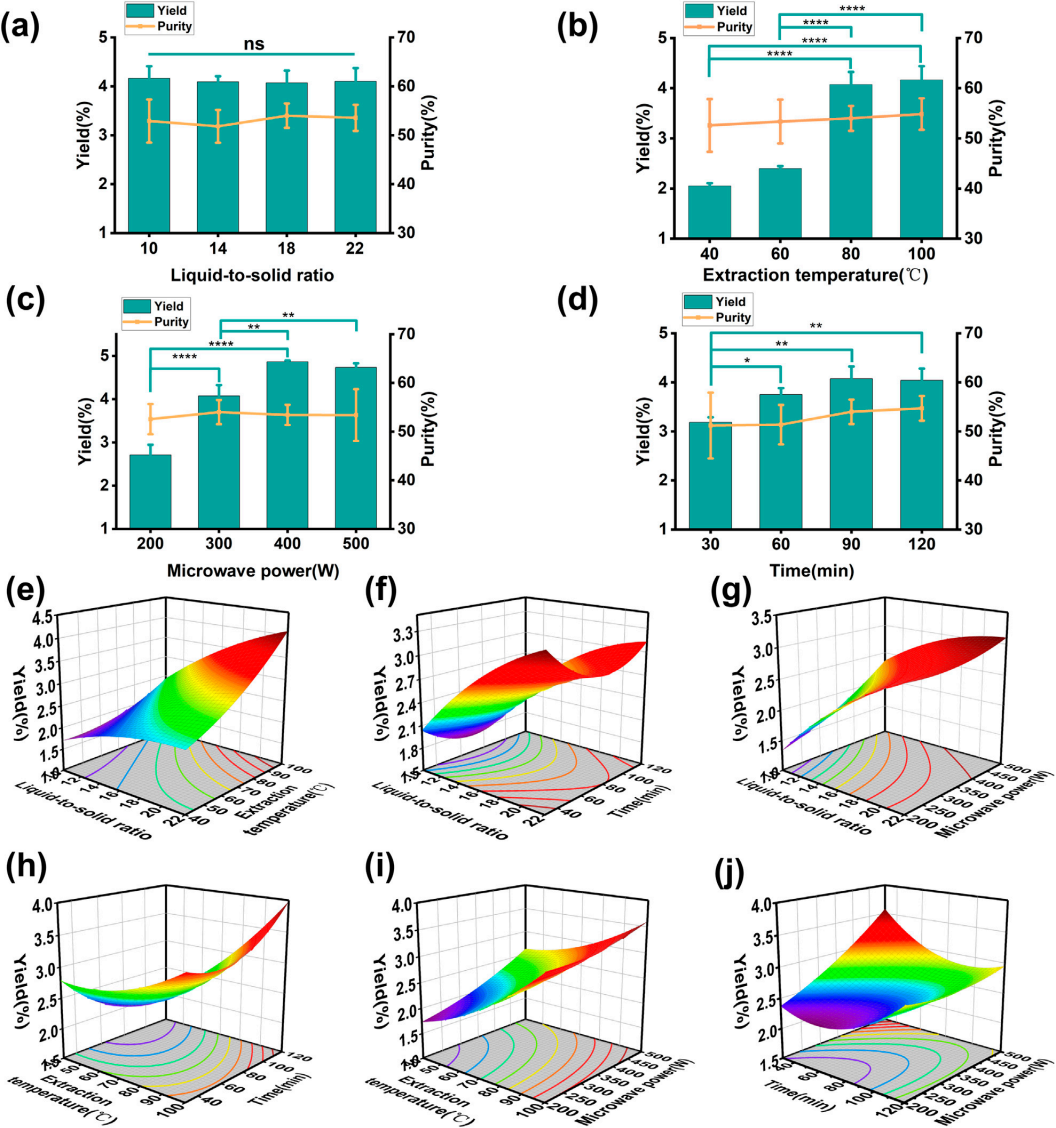
Effect of different factors on the yield of PRAC. **(A)**: liquid-to-solid ratio, **(B)**: extraction temperature, **(C)**: extraction time, **(D)**: microwave power, **(E)**: the interaction between liquid-to-solid ratio and extraction temperature, **(F)**: the interaction between liquid-to-solid ratio and time, **(G)**: the interaction between liquid-to-solid ratio and microwave power, **(H)**: the interaction between extraction temperature and time, **(I)**: the interaction between extraction temperature and microwave power, **(J)**: the interaction between time and microwave power. One-way ANOVA was used to calculate significant difference, *p < 0.05, **p < 0.01, ***p < 0.001, ****p < 0.0001.

The influence of different variables on the yield of PRAC is shown in Figures 1A–D. All variables had no significant effect on the purity of PRAC, which was about 53%. The liquid-to-solid ratio had no significant effect on polysaccharide yield, while other variables basically show an increasing first and then stabilizing trend. According to the results of single factor experiments, the scope of the various factors was determined and a Box-Behnken model was designed with four three-level factors (Table 1).

### 3.3 Box-Behnken model analysis

The Box-Behnken model was used to optimize the microwave-assisted extraction process of PRAC (Qiu J. J. et al., 2023; Qiu J. J. et al., 2024). The results of experimental and predicted values of PRAC yield are shown in Table 1. The actual yield of PRAC was between 2.149% and 4.170%. In addition, the data were fitted to the following Equation 10:

$$\begin{array}{ll} Yield= & -0.2634+0.249903\times A-0.042711\times B-0.024221\times C\\ & +0.007521\times D+0.001432\times A\times B-0.000333\times A\times C\\ & -0.00022\times A\times D+0.000246\times B\times C\\ & -0.000029\times B\times D-0.000043\times C\times D\\ & -0.004767\times A^{2}+0.00023{\times B}^{2}+0.000179\times C^{2}\\ & +4.76\times 10^{-6}\times D^{2} \end{array}$$ (10)

Where A is the liquid-to-solid ratio, B is the extraction temperature, C is the extraction time and D is the microwave power. The binomial coefficients showed that the effects of extraction temperature, extraction time and microwave power on polysaccharide yield were positively correlated, while the liquid-to-solid ratio was negatively correlated. In order to evaluate the effectiveness of the Box-Behnken model, an analysis of variance (ANOVA) was conducted (Table 2). The extraction temperature had a significant effect on the polysaccharide yield (*p*-value < 0.0001), Model *p*-value = 0.0002 < 0.05 and Lack of Fit *p*-value = 0.0527 > 0.05 to prove that the model was successfully established. R^2^ = 0.8876, adjusted R^2^ = 0.7753, and predicted R^2^ = 0.3834 were used to evaluate the fit of the model, but the low values reflected the poor fitting. The C.V. % = 7.76 indicates the reproducibility of the model. In order to clarify the relationship between four variables and PRAC yield, a 3D surface diagram (Figures 1E–J) was created. The highest PRAC yield predicted by Box-Behnken model was 4.17%, and the optimal extraction process required a 22:1 liquid-to-solid ratio, 300 W of microwave power, 100 °C of extraction temperature and 95 min of extraction time.

**TABLE 2:** ANOVE analysis of Box-Behnken model.

Source	Sum of squares	df	Mean square	F-value	*p*-value	
Model	6.41	14	0.4582	7.9	0.0002	significant
A	1.07	1	1.07	18.48	0.0007	
B	4.42	1	4.42	76.26	<0.0001	
C	0.2085	1	0.2085	3.6	0.0788	
D	0.1638	1	0.1638	2.82	0.115	
AB	0.0525	1	0.0525	0.905	0.3576	
AC	0.0064	1	0.0064	0.1102	0.7448	
AD	0.0311	1	0.0311	0.5359	0.4762	
BC	0.0868	1	0.0868	1.5	0.2413	
BD	0.0134	1	0.0134	0.2306	0.6385	
CD	0.0669	1	0.0669	1.15	0.3011	
A^2^	0.0377	1	0.0377	0.6507	0.4334	
B^2^	0.0548	1	0.0548	0.9444	0.3476	
C^2^	0.1682	1	0.1682	2.9	0.1106	
D^2^	0.0147	1	0.0147	0.2532	0.6227	
Residual	0.8119	14	0.058			
Lack of Fit	0.7594	10	0.0759	5.78	0.0527	not significant
Pure Error	0.0525	4	0.0131			
Cor Total	7.23	28				
R^2^	0.8876					
Adjusted R^2^	0.7753					
Predicted R^2^	0.3834					
C.V. %	7.76					

### 3.4 Comparation of the three kinds of artificial neural networks

Three artificial neural network models, BP, GA-BP, and GA-ACO-BP, were developed to describe the nonlinear relationship between the four input variables and polysaccharide yield. Each model has three layers: an input layer, a hidden layer and output layer. Four variables are the input layer, polysaccharide yield is the output layer, and the number of neurons in the hidden layer is determined by the equation. The optimal number of hidden layer neurons was determined by comparing the MSE of the training set as the number of hidden layer neurons varied (Hunt and Hayden, 2017).

Briefly, a BP neural network was constructed with an optimal number of hidden layer neurons of 4, a training number of 1,000. The weights and thresholds were updated each time the model was trained, and the minimum error of the training target was 0.00001. When the model reaches the number of training times or the error value between the output and the true value is less than 0.00001, the whole process stops, and the polysaccharide yield is output as the final result (Table 1).

**FIGURE 2:**
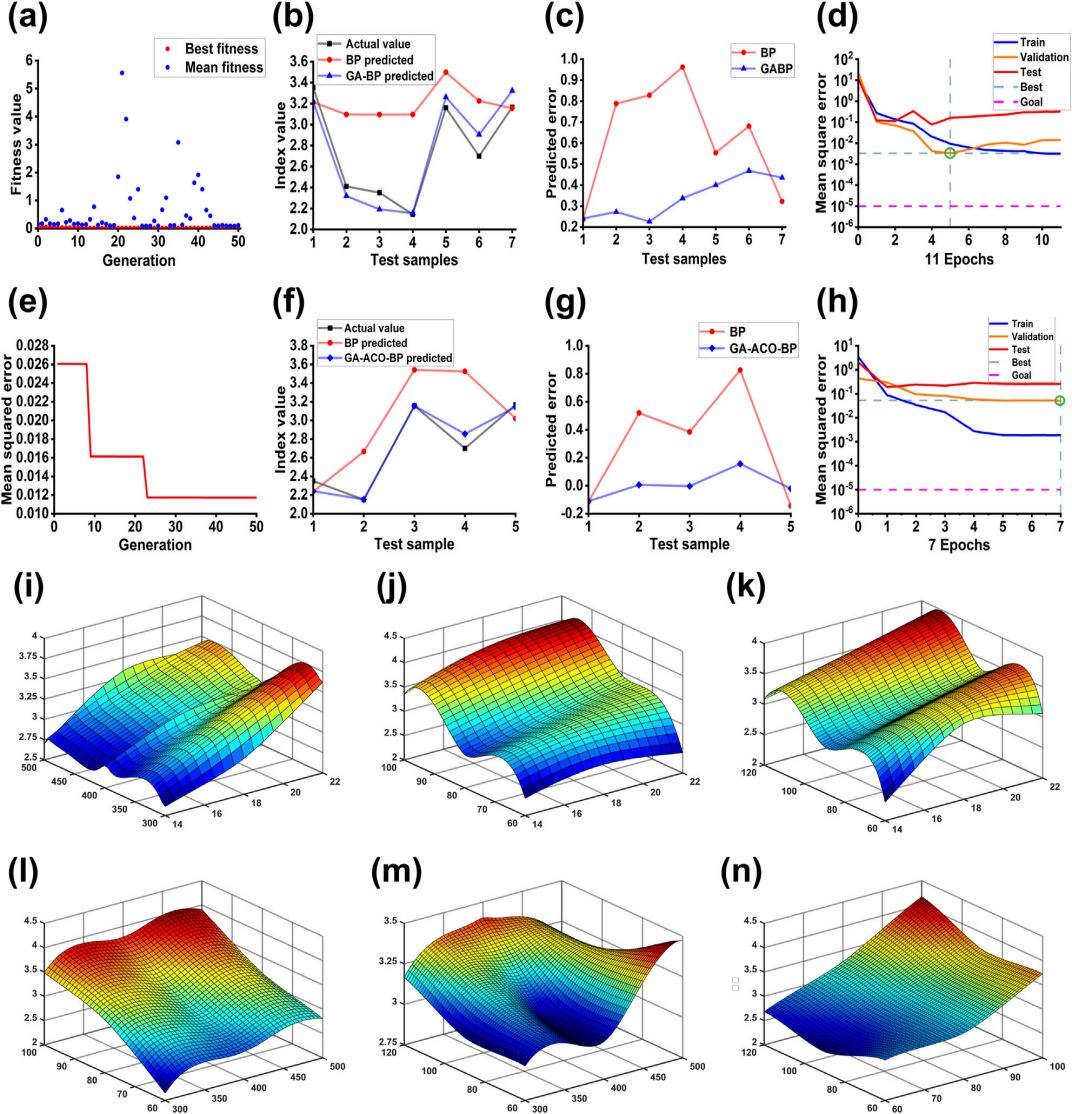
The performance of the GA-BP and GA-ACO-BP model. **(A)**: Fitness function plot of GA-BP neural network; **(B)**: The predicted values of BP neural network and GA-BP neural network were compared with the actual values; **(C)**: Comparison of predicted value error between BP neural network and GA-BP neural network; **(D)**: GA-BP neural network MSE for different data sets; **(E)**: MAE of the GA-ACO-BP model with different generation; **(F)**: The predicted values of BP neural network and GA-ACO-BP neural network were compared with the actual values; **(G)**: Comparison of predicted value error between BP neural network and GA-ACO-BP neural network; **(H)**: GA-ACO-BP neural network MSE for different data sets. **(I)**: the interaction between liquid-to-solid ratio and microwave power, **(J)**: the interaction between liquid-to-solid ratio and extraction temperature, **(K)**: the interaction between liquid-to-solid ratio and time, **(L)**: the interaction between extraction temperature and microwave power, **(M)**: the interaction between time and microwave power, **(N)**: the interaction between time and extraction temperature.

The GA-BP neural network was constructed by optimizing the threshold and weight by GA. Firstly, the parameters of the GA were initialized, with the initial population size of 30, the maximum number of iterations of 50, the crossover probability of 0.8, and the mutation probability of 0.2. The relationship between each generation and the fitness value was calculated. As shown in Figure 2A, the best fitness was 0.022, and the average fitness was 0.112. Through population selection, crossover, and mutation, the weights and thresholds were constantly updated, and finally the weights and thresholds that satisfy the convergence conditions were obtained. The optimal weights and thresholds were assigned to the BP neural network for prediction. The training times were 1,000, and the minimum error of the training target was set to 0.00001. As the number of epoch increases, the update times of the weights of the neural network also increase, and the model goes from underfitting to overfitting. Therefore, it is necessary to limit the number of epochs. It is shown in Figure 2D that the best performance of training set and test set was found at the fifth time. The highest PRAC yield predicted by GA-BP neural network was 4.32%, and the optimal extraction process is predicted as: a 22:1 liquid-to-solid ratio, 500 W of microwave power, 100°C extraction temperature and 120 min of extraction time.

The genetic algorithm was used to improve the ant colony algorithm, and the optimal weight and threshold were found to construct the GA-ACO-BP neural network (Dorigo et al., 1996). The initial population, the largest number of iterations, crossover probability and mutation probability were the same as the GA - BP neural network. The pheromone volatilization coefficient is 0.9, transition probability constant was set to 0.2, the total amount of information is 1. Then, use genetic algorithm to determine the optimal pheromones, using ant colony algorithm to determine the initial pheromone update, and then began to iteration. As the number of iterations increased, the mean square error gradually decreased, and after 23 iterations, the root mean square error tended to be stable (Figure 2E). Finally, the optimal weights and thresholds were assigned to the BP neural network, and the number of training iterations and the minimum error of the training target were set to be the same as those of the GA-BP neural network. The performance of the training set and the test set is shown in Figure 2H. The highest PRAC yield predicted by GA-ACO-BP neural network was 4.47%, and the optimal extraction process is calculated as: a 20:1 liquid-to-solid ratio, 420 W of microwave power, 100°C extraction temperature and 120 min of extraction time.

R^2^, MAE and RMSE were used to compare the predicted and true values of the four models (Jin et al., 2022; Xu et al., 2022). As shown in Table 3, the smallest MAE of 0.0632 and RMSE of 0.1139 was found in the GA-ACO-BP model, where the predicted data are the closest to the true value. On the contrary, the BP model has the largest error between the predicted value and the true value (MAE = 0.3054, RMSE = 0.4027). The calculated R^2^ of GA-ACO-BP model is 0.9480, the biggest in all models, further indicating that this model has the best predictive ability. The results of three optimal processes were verified by experiments. The yield of PRAC was 3.92% ± 0.67% in Box-Behnken model, 4.19% ± 0.52% in GA-BP model, and 4.49% ± 0.36% in GA-ACO-BP model. In conclusion, the GA-ACO-BP model is the optimal model and the predicted process is the optimal extraction process with the highest polysaccharide yield and the closest to the true value. Based on the GA-ACO-BP model, we constructed the corresponding 3D curves of the four variables and polysaccharide yield (Figures 2I–N). It can be seen that the relationship reflected by the curve is more complex than Box-Behnken model 3D graph, and possibly because the Box-Behnken is limited to the binomial regression model.

**TABLE 3:** Model performance comparison of Box-Behnken, BP neural network, GA-BP neural network and GA-ACO-BP neural network.

Model	R^2^	MAE	RMSE
Box-Behnken	0.8873	0.1492	0.1676
BP	0.3492	0.3054	0.4027
GA-BP	0.9048	0.1084	0.1540
GA-ACO-BP	0.9480	0.0632	0.1139

### 3.5 Decolorization and purification of PRAC

Polyamide adsorption was chosen as the decolorization method. The retention rate of PRAC after decolorization was 87.09% ± 1.03%, and the decolorization rate was 77.39% ± 1.36%. The purification of PRAC was carried out using anion exchange resin (Shi, 2016), The purity of PRAC was obtained as 86.53% ± 1.47%, and the loss of PRAC during the purification process was 8.27% ± 0.74%.

### 3.6 Characterization of PRAC

#### 3.6.1 Morphology of PRAC

**FIGURE 3:**
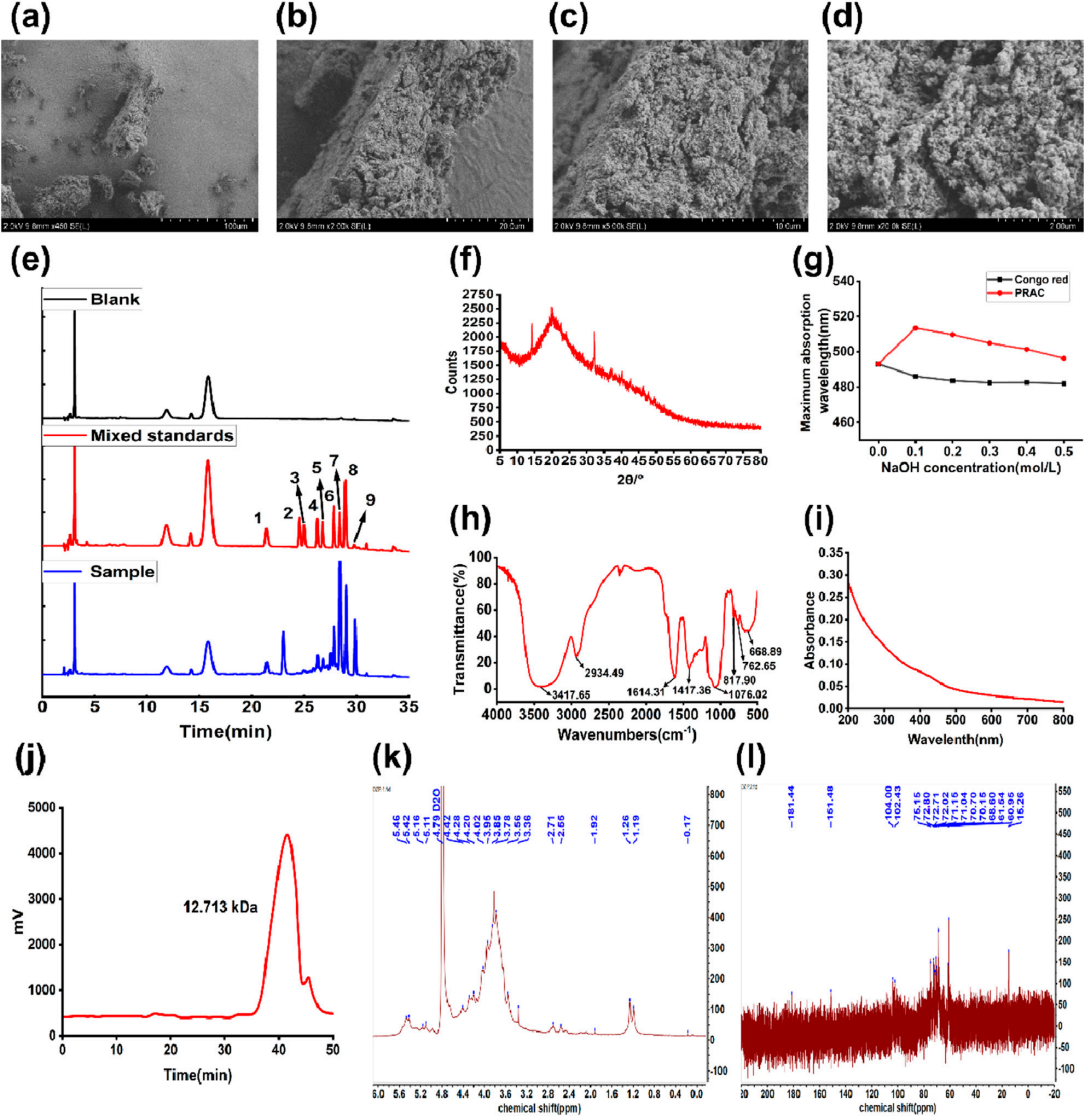
Characterization of PRAC. **(A–D)**: Scanning electron microscope images of PRAC. **(A)**:450×, **(B)**: 2000×, **(C)**: 5,000×, **(D)**: 200,00×, **(E)**: HPLC chromatograms of the monosaccharides after hydrolysis of polysaccharides. (1:D-mannose, 2:D-ribose, 3: Rhamnose, 4: D-glucuronic acid, 5: D-galacturonic acid, 6: D (+)-glucose, 7: Galactose, 8: D (+)-xylose, 9: Arabinose), **(F)**: XRD analysis of PRAC, **(G)**: Maximum UV absorption wavelength change in PRAC triple helix analysis, **(H)**: FTIR spectroscopy analysis of PRAC, **(E)**: XRD analysis of PRAC, **(I)**: PRAC UV wavelength scanning atlas, **(J)**:the GPC chromatogram of PRAC, **(K)**: ^1^H NMR spectrum of PRAC; **(L)**: ^13^C NMR spectrum of PRAC.

The PRAC were observed by SEM (Figures 3A–D). At 450× magnification, the surface of PRAC was rough and irregular shape. At ×20000 magnification, it was observed that the PRAC composed of many irregular spherical granules, and the polysaccharide structure was closely intertwined in a tight structure of a honeycomb.

#### 3.6.2 Monosaccharide composition of PRAC

The monosaccharide composition of PRAC was determined by HPLC, as shown in Figure 3E and Table 4. A total of 9 monosaccharides were detected from the chromatograms, of which galactose and xylose were the most abundant, accounting for 60.85% ± 1.40% and 14.45% ± 0.35%, respectively. It was suggested that the PRAC were mainly composed of galactose and xylose (Wu et al., 2021).

**TABLE 4:** The mole percentages of each monosaccharide in the PRAC.

Monosaccharide	Mole percent (%)
D-mannose	3.13 ± 0.89
D-ribose	0.33 ± 0.01
Rhamnose	1.18 ± 0.05
D-glucuronic acid	0.85 ± 0.37
D-galacturonic acid	1.74 ± 0.70
D (+)-glucose	8.18 ± 0.06
Galactose	60.85 ± 1.40
D (+)-xylose	14.45 ± 0.35
Arabinose	9.29 ± 3.64

#### 3.6.3 Protein and uronic acid analysis of PRAC

As shown in Figure 3I, no UV absorption between 260 nm and 280 nm were found, indicating that there were few nucleic acids and proteins in PRAC (Hu et al., 2020). In agreement with the UV spectra, the quantitative result shows that the protein content of PRAC was 0.62% ± 0.28%. This is probably due to the removal of a small amount of protein during the purification process. Uronic acid content was measured by the m-hydroxydiphenyl method, showing a content of 13.92% ± 1.83%.

#### 3.6.4 Congo red experiment

Figure 3G shows the maximum absorption wavelength of the mixed solution of PRAC and Congo red. Compared with the blank Congo red solution, there is a significant red shift at the maximum absorption wavelength after adding PRAC, indicating that PRAC can form a complex with Congo red. With the increase of NaOH concentration, the red shift effect of the disintegration of the triple helix structure decreased, and the preliminary conclusion was that PRAC had a triple helix structure (Canalejo et al., 2021).

#### 3.6.5 FTIR spectroscopy analysis

Figure 3H shows the FTIR spectra of PRAC from 500 to 4,000 cm^−1^. The sample had a strong and broad absorption peak at 3417.65 cm^−1^, which indicated the stretching vibration of the O-H bond. The absorption peak at 2934.49 cm^−1^ was attributed to C-H stretching and bending vibrations. The absorption at 1614.31 cm^−1^ represented an asymmetric stretching vibration of C=O, which may be due to the carboxyl group of uronic acid (-COOH) or the aldehyde group of aldose (-CHO) (Liu et al., 2023). The results indicated that the polysaccharide was an acidic polysaccharide containing uronic acid or aldose, which was consistent with the results of HPLC study on the monosaccharide composition of PRAC. The strong absorption peak at 1076.02 cm^-1^ was caused by the overlap of C-O-C and C-O-H stretching vibration, which was the characteristic peak of pyranoside bond, indicating that PRAC contained a pyranose ring, and the strong absorption peak at 817.90 cm^-1^ was the absorption peak of α-type glycosidic bond (Qiu J. Q. et al., 2024).

#### 3.6.6 X-ray diffraction

Figure 3F shows the X-ray diffraction results of PRAC at diffraction angles (2 θ/˚) ranging from 5˚ to 80˚. Sharp absorption peaks were observed at 14.24˚, 19.94˚ and 32.14˚, indicating the presence of a clear crystal structure in PRAC, with a small amount of absorption elsewhere, possibly with subcrystalline and amorphous structures (Jiao et al., 2023). The results of the above experiments indicated that PRAC tended to be in the semi-crystalline state. This phenomenon may be due to the change of the crystal structure of the polysaccharide in the previous purification process.

#### 3.6.7 Molecular weight analysis

The GPC chromatogram result of PRAC (Figure 3J) showed a single symmetrical peak, indicating that PRAC was relatively homogeneous and pure. Its molecular weight was determined according to the standard curve: Mw = 12.713 kDa; Mn = 9.212 kDa; and its dispersity index (Mw/Mn) was 1.38.

#### 3.6.8 Methylation analysis

Methylation analysis was performed to elucidate the glycosidic linkage between the monosaccharide units. According to the methylation results, PRAC was consisted of →4)-D-Gal*p*-(1→ (33.12%), →4)-D-Glc*p*-(1→ (28.81%), →2,3)-D-Xyl*p*-(1→ (16.48%), →4)-D-Ara*p*-(1→ (10.72%) and →4)-D-Ara*p*-(1→ (7.68%) glycosyls. These data suggest that the backbone of PRAC consists of →4)-D-Gal*p*-(1→, →4)-D-Glc*p*-(1→ and the branches are located at C2, C4, C6 of the →2,3)-D-Xyl*p*-(1→, →4)-D-Ara*p*-(1→, →4)-D-Ara*p*-(1→.

#### 3.6.9 Nuclear magnetic resonance

The ^1^H NMR spectrum of PRAC is shown in Figure 3K. Signal peaks appeared at 5.46 ppm, 5.42 ppm, 4.42 ppm, and 4.28 ppm, indicating the presence of both α-glycosidic bonds and β-glycosidic bonds in PRAC. The other signals are mainly concentrated at 3.2–4.5 ppm, which is mainly due to the H-2 to H-6 protons on the glycosidic ring (Shang et al., 2021).

As shown in Figure 3L, the anomeric carbon signals of PRAC were concentrated in the region from 90 to 110 ppm. There were two more obvious signals in this region, which reflected that PRAC has a complex glycosidic bond linkage. Combining with infrared spectrum analysis, it could be inferred that the glycosidic bond in PRAC was α-D type. The region between δ 50 and δ 90 is the distribution region of the C2 to C6 signals of monosaccharide residues. The signals at around δ 60 were attributed to the C6 signal of the hexose. The strong peak at δ 15.26 in the high field region was attributed to the signal of the primary carbon, which indicated the presence of deoxygenated sugar residues in the structure of PRAC. Based on previous studies, this signal was inferred to be the C6 signal of rhamnose (Lin and Huang, 2022). In the low-field region, δ 181.44 was the chemical shift of carbonyl group (-CO-) on the sugar ring, which is consistent with the results of infrared spectroscopy.

### 3.7 Characterization of PRAC-loaded liposomes

**FIGURE 4:**
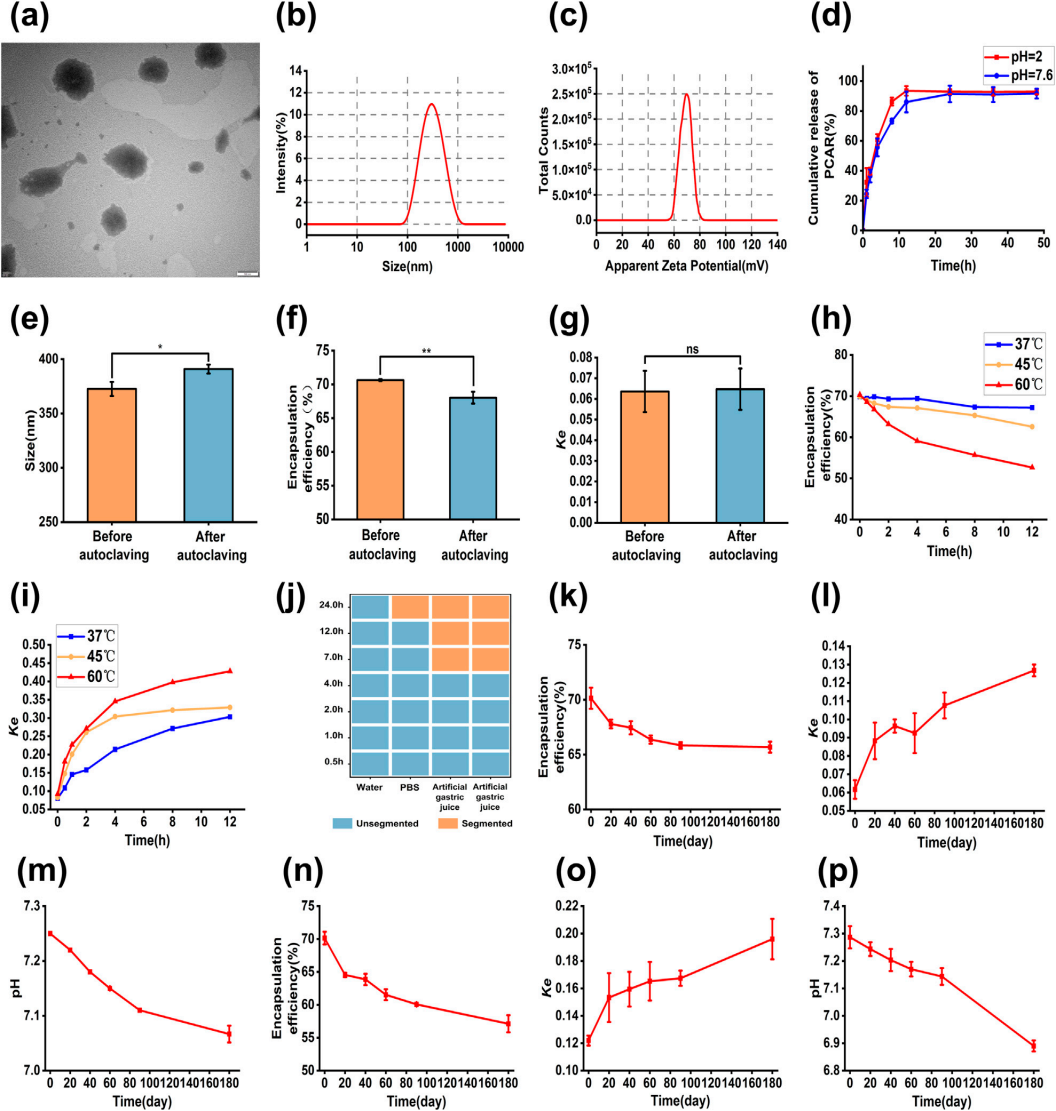
Characterization results of PRAC-loaded liposomes. **(A)**: TEM images of liposome, **(B)**: Particle size of liposome, **(C)**: Zeta potential of liposome, **(D)**: The cumulative release kinetics of PRAC from liposome, **(E)**: Changes in particle size of liposomes before and after autoclaving, **(F)**: Changes in the encapsulation efficiency of liposomes before and after autoclaving, **(G)**: Changes in the *Ke* of liposomes before and after autoclaving, **(H)**: Changes in the encapsulation efficiency of liposomes at different temperature environments, **(I)**: Changes in the *Ke* of liposomes at different temperature environments, **(J)**: Stability of liposomes in different media, **(K)**: Change in encapsulation efficiency at 4°C, **(L)**: Change in *Ke* at 4°C, **(M)**: Change in pH at 4°C, **(N)**: Change in encapsulation efficiency at 25°C, **(O)**: Change in *Ke* at 25°C, **(P)**: Change in pH at 25°C. Student’s t tests were used to calculate significant difference, *p < 0.05, **p < 0.01.

The Dynamic Light Scattering (DLS) detection results of the PRAC-loaded liposomes were shown in Figures 4A–C. The particle size of the liposomes was 342.9 ± 13.7 nm, PDI was 0.234 ± 0.1, and Zeta potential was-61.3 ± 1.63 mV. The TEM showed a slightly smaller particle size than the DLS results, mainly due to the liposome crumpling when the TEM was filmed in a dry environment. The pH of the liposomes was 7.21 ± 0.04. The encapsulation efficiency of the liposomes was 70.12% ± 0.98% and the drug loading was 1.24% ± 0.02%. The stability coefficient *Ke* was 0.0670 ± 0.51.

### 3.8 *In vitro* release of PRAC-loaded liposomes

The cumulative release of PRAC-loaded liposomes *in vitro* is shown in Figure 4D. In different pH environments, the release of liposomes conformed to the first-order kinetic equation as following Equations 11, 12.

$$pH=2,M_{t}=93.47\left(1-e^{-0.30t}\right),R^{2}=0.9882$$ (11)

$$pH=7.6,M_{t}=90.68\left(1-e^{-0.24t}\right),R^{2}=0.9939$$ (12)

Liposomes exhibited a release of 60.84% in pH = 2 PBS solution and 55.65% in pH = 7.6 PBS solution within 4 h. At 12 h, the cumulative release of the liposomes was 93.45% in pH = 2 PBS solution, and 86.00% in pH = 7.6 PBS solution, respectively. Human gastrointestinal emptying time is about 4–6 h, so liposomes can effectively reduce the exposure time of PRAC in the gastrointestinal tract and reduce the structural damage of PRAC.

### 3.9 Preliminary stability of PRAC-loaded liposomes

The results show that after autoclave sterilization, the PRAC-loaded liposomes changed from milky white to yellowish and were uniformly dispersed without stratification. However, the particle size and encapsulation efficiency of the PRAC-loaded liposomes changed significantly (Figures 4E–G). This indicated that the PRAC-loaded liposomes could not remain stable after autoclaving.

The encapsulation efficiency of liposomes at 37°C was no significant difference within 4 h (Figures 4H, I). With the increase of incubation time, the encapsulation efficiency of liposomes gradually decreased, and the stability coefficient also increased, indicating that the temperature could accelerate the condensation and leakage of liposomes and reduce the stability of liposomes. At 45°C and 60°C, the encapsulation efficiency and stability coefficient of the liposomes changed significantly, indicating that the liposomes were not suitable for long-term storage at high temperature.

There were differences in the stability of the liposomes in different media (Figure 4J). There was no significant change in ultrapure water, which could be used as the dispersion medium of liposomes. It was stable in PBS for 12 h, but delamination occurred after 24 h, and dispersed into uniform solution after shaking. Stability reduced greatly in artificial gastric and intestinal fluids, and after 7 h, no homogeneous solution could be formed.

### 3.10 The long-term stability of the PRAC-loaded liposomes

There was no significant change in the encapsulation efficiency, stability coefficient and pH value of the liposomes after 6 months of storage at 4°C (Figures 4K–P). The results indicated that the liposome solution had good stability and 4°C was suitable for long-term storage of the liposome solution. However, the particle size and Zeta potential of the liposomes gradually increased at 25°C and could not be detected at 180 days. And at the same time, liposome encapsulation efficiency decreased, the stability coefficient increased, and the pH gradually decreased, but the polysaccharide content in the liposomes basically remained unchanged.

### 3.11 Therapeutic effect of PRAC-loaded liposomes on acute liver injury

Liver injury often occurs for a variety of reasons involving viruses, inflammatory mediator release, immune dysfunction, and free radical damage. Therefore, in this study, we established CCl_4_ acute liver injury model (Taniguchi et al., 2004), D-gal acute liver injury model (Xie et al., 2022) and ConA immune liver injury model (Erhardt et al., 2007) to study the therapeutic effect of PRAC-loaded liposomes on liver injury.

**FIGURE 5:**
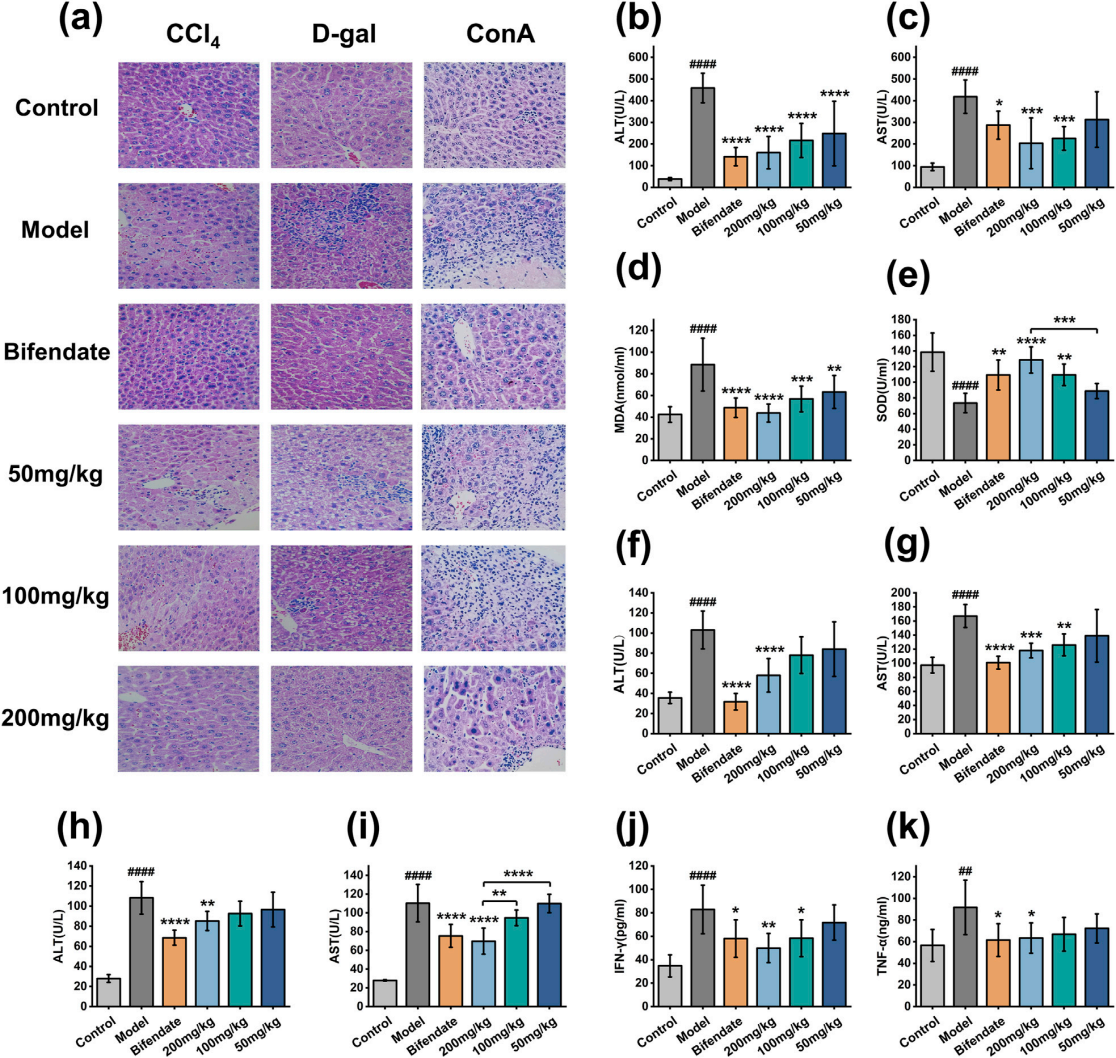
Therapeutic effect of PRAC-loaded liposomes on acute liver injury. **(A)**:H&E images of mouse liver tissue (200×), **(B–E)**: The levels of ALT, AST, MDA and SOD in CCl_4_ mice acute liver injury experiment, **(F, G)**: ALT and AST levels in each group of D-gal mice in the acute liver injury experiment, **(H–K)**: ConA immune liver injury in mice experiment of ALT, AST, IFN-γ and TNF-ɑ levels. Data are presented as means ± SD (n = 12). Multi-way ANOVA was used to calculate significant difference, Control vs. Model ####*p* < 0.0001, ##*p* < 0.01; Each administration group vs. Model *p < 0.05, **p < 0.01, ***p < 0.001, ****p < 0.0001.

Figures 5B–E shows the serum levels of the ALT, AST, MDA and SOD in each group of mice in CCl_4_ acute liver injury experiment. Compared with the control group, ALT, AST and MDA in the model group were significantly increased, and SOD was significantly decreased, indicating that the model was successfully established. Compared with the model group, ALT, AST and MDA levels were significantly decreased and SOD was significantly increased in the 200 mg/kg and 100 mg/kg groups. There was no significant difference in AST and SOD between the 50 mg/kg group and the model group. In the D-gal acute liver injury experiment (Figures 5F, G), compared with the control group, ALT and AST in the model group were significantly increased, indicating that the model was successfully established. Compared with model group, 200 mg/kg and 100 mg/kg group of ALT and AST were significantly reduced with no significant difference in the 50 mg/kg group. In the ConA immune liver injury experiment (Figures 5H–K), compared with the control group, ALT, AST, IFN-γ, and TNF-ɑ in the model group were significantly increased, indicating that the model was successfully established. Compared with the model group, the levels of ALT, AST, IFN-γ and TNF-ɑ were significantly decreased in the 200 mg/kg group, but there was no significant difference in the 100 mg/kg and 50 mg/kg groups.

H&E showed (Figure 5A) that in the control group, the hepatic lobules were arranged neatly, a few blood vessels showed very mild peritubular infiltration. In the model group, most of the hepatocytes were swollen and mild steatosis. Most of the blood vessels showed moderate peritubular infiltration, large area of focal necrosis, inflammatory cell infiltration, and Kupffer’s cells increased. In 200 mg/kg, 100 mg/kg and bifendate groups, scattered punctate necrosis and focal necrosis were observed in some liver tissues, and the degree of liver cell injury was significantly reduced, and the infiltration of inflammatory cells was significantly reduced (Yin et al., 2007). There was no significant improvement in the degree of liver tissue lesions in the 50 mg/kg group. In conclusion, PRAC-loaded liposomes have a therapeutic effect on acute liver injury in a dose-dependent manner.

## 4 Discussion

The Box-Behnken model is used to describe the relationship between input and output variables by building a mathematical model, the exact implementation of which depends on the type of experimental optimization. However, when the number of input variables increases, the results of the Box-Behnken model show a poor fit, which leads to problems such as insignificant modeling, poor fit, and non-significant fit. Artificial neural network is a computational model that mimics a biological nervous system and consists of many simple neurons that can be used for a variety of tasks such as pattern recognition, classification, prediction and optimization (Xie et al., 2015). Artificial neural networks can process a large amount of data efficiently, learn from a large amount of data, and constantly change their own weights and thresholds to gradually improve their fitting and prediction ability, which is very helpful for explaining some nonlinear problems; moreover, when the problem changes, artificial neural networks can adjust their structure and parameters to adapt to different environments according to the actual situation (Bond-Taylor et al., 2022; Lake and Baroni, 2023). In this experiment, three artificial neural networks were trained to fit the polysaccharide extraction process. The fitting and prediction ability of three neural networks with the Box-Behnken model was evaluated using the metrics R^2^, RMSE and MAE. The GA-ACO-BP model was found to outperform the other models in terms of fitting and prediction when the extraction process was investigated, and the highest yield of polysaccharides was obtained from the optimal process of this model. The 3D plots generated by the Box-Behnken model showed a single trend between the output and input variables, while the 3D plots generated by the GA-ACO-BP model showed a more complex relationship between the output and input variables. However, artificial neural networks have several drawbacks, such as the need for a large amount of training data, and therefore, they cannot be used effectively with a very small number of samples. When there are too many parameters in the model, overfitting tends to occur, leading to a decrease in the model’s generalization ability. In addition, when the fit is inadequate, it is easy to find local optimal solutions. The present work demonstrates that despite some important remaining hurdles, combining the latest developments in artificial neural network offers exciting avenues for drug chemistry and engineering. Soon, these tools may provide solutions in quality control and raw material development.

Polysaccharides are polymers formed by the condensation of several monosaccharide molecules, which are composed of primary structure and higher structure. The primary structure mainly includes the composition of monosaccharide residues, the connection mode and the glycosidic bond between monosaccharides, etc. (Chen et al., 2022). The current research also focuses on the analysis of the primary structure of polysaccharides. Different structures of polysaccharides are inextricably linked to their pharmacological activities. Although it is difficult to clarify the structure–activity correlations of polysaccharides, some possible relationships can be deduced (Ferreira et al., 2015). In this study, a galactan with an average molecular weight of 12.713 kDa, named PRAC, was isolated from *Radix Actinidiae Chinensis*. Its structure consisted of a repeating →4)-D-Gal*p*-(1→ and →4)-D-Glc*p*-(1→ unit as a main chain with branching points occurring at the C2 C4, and C6, which were replaced by various types of Xyl*p* and Ara*p* side chains to form multibranches. XRD was used to analyze the crystal structure of the polysaccharide. PRAC is mainly present in amorphous form, which is also a common feature of polysaccharides. The structure of PRAC is affected by the source, growth environment, extraction process and purification process, which is one of the main factors limiting the study of polysaccharide bioactivities, future studies will focus on controlling a single variable to examine the structure of polysaccharides (Xu et al., 2017).

PRAC are macromolecular polysaccharides, preliminary laboratory studies have shown poor efficacy for oral administration. The drug delivered orally will first enter the gastrointestinal environment, which has many biological and physical obstacles that prevent the drug from being absorbed into the blood circulation smoothly (Large et al., 2021; Zahednezhad et al., 2019). In the present study, PRAC were maximally loaded in liposomes for effective delivery of polysaccharides. The prepared PRAC liposomes had a particle size of about 340 nm, a PDI of 0.234, a zeta potential of −61.3 mV, an entrapment efficiency of 70.12% and a drug loading capacity of 1.24%. PRAC was released completely from the liposome within 12 h. It can increase the stability of PRAC in the gastrointestinal tract and avoid hydrolysis by biological metabolic enzymes such as pepsin and amylase. Due to the amphiphilic nature of liposomes, it may be possible to increase the absorption of PRAC by small intestinal epithelial cells and increase the bioavailability of PRAC (He H. S. et al., 2019; Manconi et al., 2020; Zou et al., 2024).

Radix Actinidiae Chinensis have antibacterial, anti-inflammatory and antitumor pharmacological effects, and there are also studies on liver protection, but relatively few. In this study, it was found that PRAC-loaded liposomes had a significant therapeutic effect on acute liver injury at a dose of 100 mg/kg. PRAC can downregulate the levels of ALT, AST and MDA and upregulate the level of SOD, thus playing a therapeutic role in liver injury. On the other hand, we validated the therapeutic effect of PRAC on non-alcoholic acute liver injury in three different animal models. It is of great practical significance to establish animal models with different injury mechanisms, comprehensively determine the efficacy and mechanism of drugs and screen hepatoprotective drugs for the treatment of acute liver injury (Michalopoulos and Bhushan, 2021). Based on the above findings, PRAC may be a potential drug for clinical treatment of acute liver injury, but further investigation is essential to elucidate its mechanism of action more clearly.

## Data Availability

The raw datasets presented in this study are stored in the Figshare Dryad Digital Repository; data link: https://doi.org/10.6084/m9.figshare.28400054.

## Glossary

PRAC: polyacrylamide of Radix Actinidiae Chinensis
RAC: Radix Actinidiae Chinensis
BP: back propagation
GA: Genetic algorithm
ACO: Ant Colony Optimization
SEM: scanning electron microscopy
FTIR: fourier transform infrared spectroscopy
HPLC: high performance liquid chromatography
UV: ultraviolet spectroscopy
XRD: X-ray diffraction
NMR: nuclear magnetic resonance
GPC: gel permeation chromatography
GC-MS: gas chromatography–mass spec-trometry
CR mice: institute of cancer research mouse
R^2^: coefficient of determination
MSE: set of mean square error
MAE: mean absolute error
RMSE: root mean square error
TFA: trifluoroacetic acid
PMP: 3-Methyl-1-phenyl-2-pyrazolin-5-one
PEG-2000: polyethylene glycol 2000
TEM: transmission electron microscopy
PDI: polymer dispersity index
LC: loading capacity
EE: encapsulation efficiency
PBS: phosphate buffered saline
ALT: alanine aminotransferase
AST: aspertate aminotransferase
SOD: superoxide dismutase
MDA: malondialdehyde
D-gal: D-galactosamine
ConA: concanavalin protein
SD: standard deviation
ANOVA: one-way analysis of variance.

## References

[B1] Badhwar P. Kumar A. Yadav A. Kumar P. Siwach R. Chhabra D. (2020). Improved pullulan production and process optimization using novel GA-ANN and GA-ANFIS hybrid statistical tools. Biomolecules 10 (1), 124. 10.3390/biom10010124 31936881 PMC7022329

[B2] Bagade S. B. Patil M. (2021). Recent advances in microwave assisted extraction of bioactive compounds from complex herbal samples: a review. Crit. Rev. Anal. Chem. 51 (2), 138–149. 10.1080/10408347.2019.1686966 31729248

[B3] Bond-Taylor S. Leach A. Long Y. Willcocks C. G. (2022). Deep generative modelling: a comparative review of VAEs, GANs, normalizing flows, energy-based and autoregressive models. Ieee Trans. Pattern Analysis Mach. Intell. 44 (11), 7327–7347. 10.1109/TPAMI.2021.3116668 34591756

[B4] Canalejo D. Guadalupe Z. Martínez-Lapuente L. Ayestarán B. Pérez-Magariño S. (2021). Optimization of a method to extract polysaccharides from white grape pomace by-products. Food Chem. 365, 130445. 10.1016/j.foodchem.2021.130445 34237579

[B5] Chen L. Long R. Huang G. L. Huang H. L. (2020). Extraction and antioxidant activities *in vivo* of pumpkin polysaccharide. Industrial Crops Prod. 146, 112199. 10.1016/j.indcrop.2020.112199

[B6] Chen X. X. Yang J. Shen M. Y. Chen Y. Yu Q. Xie J. H. (2022). Structure, function and advance application of microwave-treated polysaccharide: a review. Trends Food Sci. and Technol. 123, 198–209. 10.1016/j.tifs.2022.03.016

[B8] Dorigo M. Maniezzo V. Colorni A. (1996). Ant system: optimization by a colony of cooperating agents. IEEE Trans. Syst. man, Cybern. 26 (1), 29–41. 10.1109/3477.484436 18263004

[B9] Duvvuri S. P. Anmala J. (2019). Fecal coliform predictive model using genetic algorithm-based radial basis function neural networks (GA-RBFNNs). Neural Comput. and Appl. 31 (12), 8393–8409. 10.1007/s00521-019-04520-2

[B10] Erhardt A. Biburger M. Papadopoulos T. Tiegs G. (2007). IL-10, regulatory T cells, and Kupffer cells mediate tolerance in concanavalin A-induced liver injury in mice. Hepatology 45 (2), 475–485. 10.1002/hep.21498 17256743

[B11] Fang T. T. Zhao Z. Y. Yuan F. F. He M. Y. Sun J. L. Guo M. Z. (2020). *Actinidia Chinensis* Planch Root extract attenuates proliferation and metastasis of hepatocellular carcinoma by inhibiting the DLX2/TARBP2/JNK/AKT pathway. J. Ethnopharmacol. 251, 112529. 10.1016/j.jep.2019.112529 31891797

[B12] Ferreira S. S. Passos C. P. Madureira P. Vilanova M. Coimbra M. A. (2015). Structure function relationships of immunostimulatory polysaccharides: a review. Carbohydr. Polym. 132, 378–396. 10.1016/j.carbpol.2015.05.079 26256362

[B13] He H. S. Lu Y. Qi J. P. Zhu Q. G. Chen Z. J. Wu W. (2019a). Adapting liposomes for oral drug delivery. Acta Pharm. Sin. B 9 (1), 36–48. 10.1016/j.apsb.2018.06.005 30766776 PMC6362257

[B14] He X. R. Fang J. C. Chen X. F. Zhao Z. F. Li Y. S. Meng Y. B. (2019b). *Actinidia chinensis* planch.: a review of chemistry and pharmacology. Front. Pharmacol. 10, 1236. 10.3389/fphar.2019.01236 31736750 PMC6833939

[B15] Heya M. S. García-Ponce R. Soto B. A. M. Verde-Star M. J. Soto-Domínguez A. García-Hernandez D. G. (2023). Green alternatives in treatment of liver diseases: the challenges of traditional medicine and green nanomedicine. Nanomedicine. Chem. and Biodivers. 20, e202300463. 10.1002/cbdv.202300463 37531499

[B16] Hosack T. Damry D. Biswas S. (2023). Drug-induced liver injury: a comprehensive review. Ther. Adv. Gastroenterology 16, 17562848231163410. 10.1177/17562848231163410 PMC10031606 36968618

[B17] Hu J. L. Liu Y. Cheng L. M. Shi R. J. Qayum A. Bilawal A. (2020). Comparison in bioactivity and characteristics of *Ginkgo biloba* seed polysaccharides from four extract pathways. Int. J. Biol. Macromol. 159, 1156–1164. 10.1016/j.ijbiomac.2020.05.129 32442575

[B18] Hu Z. Hong P. Z. Cheng Y. Liao M. N. Li S. D. (2018). Polysaccharides from *Enteromorpha tubulosa*: optimization of extraction and cytotoxicity. J. Food Process. Preserv. 42 (1), e13373. 10.1111/jfpp.13373

[B19] Huang G. L. Chen F. Yang W. J. Huang H. L. (2021). Preparation, deproteinization and comparison of bioactive polysaccharides. Trends Food Sci. and Technol. 109, 564–568. 10.1016/j.tifs.2021.01.038

[B20] Hunt L. T. Hayden B. Y. (2017). A distributed, hierarchical and recurrent framework for reward-based choice. Nat. Rev. Neurosci. 18 (3), 172–182. 10.1038/nrn.2017.7 28209978 PMC5621622

[B21] Irani R. Nasimi R. (2011). Evolving neural network using real coded genetic algorithm for permeability estimation of the reservoir. Expert Syst. Appl. 38 (8), 9862–9866. 10.1016/j.eswa.2011.02.046

[B22] Jiao X. Li F. Zhao J. Wei Y. L. Zhang L. Y. Wang H. J. (2023). Structural diversity and physicochemical properties of polysaccharides isolated from pumpkin (*Cucurbita moschata*) by different methods. Food Res. Int. 163, 112157. 10.1016/j.foodres.2022.112157 36596108

[B23] Jin L. Jin W. F. Zhang Y. Y. Xu S. C. Wan H. T. He Y. (2022). Simultaneous optimization of the extraction process of Yangyin Yiqi Huoxue prescription with natural deep eutectic solvents for optimal extraction yield and antioxidant activity: a comparative study of two models. Phytomedicine 102, 154156. 10.1016/j.phymed.2022.154156 35550223

[B24] Lake B. M. Baroni M. (2023). Human-like systematic generalization through a meta-learning neural network. Nature 623 (7985), 115–121. 10.1038/s41586-023-06668-3 37880371 PMC10620072

[B25] Large D. E. Abdelmessih R. G. Fink E. A. Auguste D. T. (2021). Liposome composition in drug delivery design, synthesis, characterization, and clinical application. Adv. Drug Deliv. Rev. 176, 113851. 10.1016/j.addr.2021.113851 34224787

[B26] Lefere S. Puengel T. Hundertmark J. Penners C. Frank A. K. Guillot A. (2020). Differential effects of selective- and pan-PPAR agonists on experimental steatohepatitis and hepatic macrophages. J. Hepatology 73 (4), 757–770. 10.1016/j.jhep.2020.04.025 32360434

[B27] Leong Y. K. Yang F. C. Chang J. S. (2021). Extraction of polysaccharides from edible mushrooms: emerging technologies and recent advances. Carbohydr. Polym. 251, 117006. 10.1016/j.carbpol.2020.117006 33142573

[B28] Liang J. Wang W. F. Zhang Y. Chai Y. Q. Li Y. G. Jiang S. L. (2024). Fructooligosaccharides and fructans from *Platycodon grandiflorum*: structural characterization, lung-oriented guidance and targetability. Carbohydr. Polym. 323, 121457. 10.1016/j.carbpol.2023.121457 37940316

[B29] Lin B. B. Huang G. L. (2022). An important polysaccharide from fermentum. Food Chemistry-X 15, 100388. 10.1016/j.fochx.2022.100388 36211774 PMC9532711

[B30] Liu F. Chen H. J. Qin L. Al-Haimi A. Xu J. Zhou W. Z. (2023). Effect and characterization of polysaccharides extracted from *Chlorella* sp. by hot-water and alkali extraction methods. Algal Research-Biomass Biofuels Bioprod. 70, 102970. 10.1016/j.algal.2023.102970

[B31] Loomba R. Friedman S. L. Shulman G. I. (2021). Mechanisms and disease consequences of nonalcoholic fatty liver disease. Cell 184 (10), 2537–2564. 10.1016/j.cell.2021.04.015 33989548 PMC12168897

[B32] Manconi M. Caddeo C. Manca M. L. Fadda A. M. (2020). Oral delivery of natural compounds by phospholipid vesicles. Nanomedicine 15 (18), 1795–1803. 10.2217/nnm-2020-0085 32698672

[B33] Michalopoulos G. K. Bhushan B. (2021). Liver regeneration: biological and pathological mechanisms and implications. Nat. Rev. Gastroenterology and Hepatology 18 (1), 40–55. 10.1038/s41575-020-0342-4 32764740

[B34] Ong H. C. Milano J. Silitonga A. S. Hassan M. H. Shamsuddin A. H. Wang C. T. (2019). Biodiesel production from *Calophyllum inophyllum-Ceiba pentandra* oil mixture: optimization and characterization. J. Clean. Prod. 219, 183–198. 10.1016/j.jclepro.2019.02.048

[B35] Parlati L. Régnier M. Guillou H. Postic C. (2021). New targets for NAFLD. Jhep Rep. 3 (6), 100346. 10.1016/j.jhepr.2021.100346 34667947 PMC8507191

[B36] Picot-Allain C. Mahomoodally M. F. Ak G. Zengin G. (2021). Conventional versus green extraction techniques - a comparative perspective. Curr. Opin. Food Sci. 40, 144–156. 10.1016/j.cofs.2021.02.009

[B37] Qiu J. J. Shi M. L. Li S. Q. Ying Q. Y. Zhang X. X. Mao X. X. (2023a). Artificial neural network model- and response surface methodology-based optimization of Atractylodis Macrocephalae Rhizoma polysaccharide extraction, kinetic modelling and structural characterization. Ultrason. Sonochemistry 95, 106408. 10.1016/j.ultsonch.2023.106408 PMC10457599 37088027

[B38] Qiu J. J. Xu X. Guo J. Y. Wang Z. Y. Wu J. J. Ding H. Q. (2024a). Comparison of extraction processes, characterization and intestinal protection activity of Bletilla striata polysaccharides. Int. J. Biol. Macromol. 263, 130267. 10.1016/j.ijbiomac.2024.130267 38378109

[B39] Qiu J. Q. Zheng P. Y. Dai W. Z. Zheng Z. J. Lin X. H. Hu J. M. (2024b). Steam explosion-assisted extraction of polysaccharides from *Pleurotus eryngii* and its influence on structural characteristics and antioxidant activity. Foods 13 (8), 1229. 10.3390/foods13081229 38672901 PMC11049414

[B40] Qiu K. J. Xia Q. Chen H. Ye Q. Mao H. X. Tian M. (2023b). Exploring the anticancer potential of Actinidia chinensis Planch root extracts (*acRoots*) on hepatocellular carcinoma: a molecular mechanism study. Heliyon 9 (11), e21851. 10.1016/j.heliyon.2023.e21851 38027882 PMC10656260

[B41] Rathore A. S. Nikita S. Thakur G. Mishra S. (2023). Artificial intelligence and machine learning applications in biopharmaceutical manufacturing. Trends Biotechnol. 41 (4), 497–510. 10.1016/j.tibtech.2022.08.007 36117026

[B42] Riaz M. Khalid R. Afzal M. Anjum F. Fatima H. Zia S. (2023). Phytobioactive compounds as therapeutic agents for human diseases: a review. Food Sci. and Nutr. 11 (6), 2500–2529. 10.1002/fsn3.3308 37324906 PMC10261751

[B43] Shang X. C. Chu D. P. Zhang J. X. Zheng Y. F. Li Y. Q. (2021). Microwave-assisted extraction, partial purification and biological activity *in vitro* of polysaccharides from bladder-wrack (*Fucus vesiculosus*) by using deep eutectic solvents. Sep. Purif. Technol. 259, 118169. 10.1016/j.seppur.2020.118169

[B44] Shi L. (2016). Bioactivities, isolation and purification methods of polysaccharides from natural products: a review. Int. J. Biol. Macromol. 92, 37–48. 10.1016/j.ijbiomac.2016.06.100 27377457 PMC7124366

[B45] Tang Y. Y. He X. M. Liu G. M. Wei Z. Sheng J. F. Sun J. (2023). Effects of different extraction methods on the structural, antioxidant and hypoglycemic properties of red pitaya stem polysaccharide. Food Chem. 405, 134804. 10.1016/j.foodchem.2022.134804 36356363

[B46] Taniguchi M. Takeuchi T. Nakatsuka R. Watanabe T. Sato K. (2004). Molecular process in acute liver injury and regeneration induced by carbon tetrachloride. Life Sci. 75 (13), 1539–1549. 10.1016/j.lfs.2004.02.030 15261760

[B47] Tu Y. J. Luo X. Y. Liu D. Li H. J. Xia H. Y. Ma C. Z. (2022). Extracts of *Poria cocos* improve functional dyspepsia via regulating brain-gut peptides, immunity and repairing of gastrointestinal mucosa. Phytomedicine 95, 153875. 10.1016/j.phymed.2021.153875 34911003

[B48] Wang Y. J. Xiong X. Huang G. L. (2023). Ultrasound-assisted extraction and analysis of maidenhairtree polysaccharides. Ultrason. Sonochemistry 95, 106395. 10.1016/j.ultsonch.2023.106395 PMC10439246 37015179

[B49] Wu D. T. Zhao Y. X. Guo H. Gan R. Y. Peng L. X. Zhao G. (2021). Physicochemical and biological properties of polysaccharides from *Dictyophora indusiata* prepared by different extraction techniques. Polymers 13 (14), 2357. 10.3390/polym13142357 34301113 PMC8309502

[B50] Xie R.-F. Shi Z.-N. Li Z.-C. Chen P.-P. Li Y.-M. Zhou X. (2015). Optimization of high pressure machine decocting process for Dachengqi Tang using HPLC fingerprints combined with the Box-Behnken experimental design. J. Pharm. analysis 5 (2), 110–119. 10.1016/j.jpha.2014.07.001 PMC5761470 29403922

[B51] Xie Y. H. Li J. Qin H. Wang Q. Chen Z. X. Liu C. Y. (2022). Paramylon from *Euglena gracilis* prevents lipopolysaccharide-induced acute liver injury. Front. Immunol. 12, 797096. 10.3389/fimmu.2021.797096 35126359 PMC8812190

[B52] Xu H. S. Wu Y. W. Xu S. F. Sun H. X. Chen F. Y. Yao L. (2009). Antitumor and immunomodulatory activity of polysaccharides from the roots of *Actinidia eriantha* . J. Ethnopharmacol. 125 (2), 310–317. 10.1016/j.jep.2009.06.015 19559777

[B53] Xu S. Wan H. Zhao X. Zhang Y. Yang J. Jin W. (2022). Optimization of extraction and purification processes of six flavonoid components from Radix Astragali using BP neural network combined with particle swarm optimization and genetic algorithm. Industrial Crops Prod. 178, 114556. 10.1016/j.indcrop.2022.114556

[B54] Xu S. Y. Huang X. S. Cheong K. L. (2017). Recent advances in marine algae polysaccharides: isolation, structure, and activities. Mar. Drugs 15 (12), 388. 10.3390/md15120388 29236064 PMC5742848

[B55] Yang Y. M. Cho Y. E. Hwang S. (2022). Crosstalk between oxidative stress and inflammatory liver injury in the pathogenesis of alcoholic liver disease. Int. J. Mol. Sci. 23 (2), 774. 10.3390/ijms23020774 35054960 PMC8775426

[B56] Yin H. Cheng L. Langenbach R. Ju C. (2007). Prostaglandin I(2) and E(2) mediate the protective effects of cyclooxygenase-2 in a mouse model of immune-mediated liver injury. Hepatology 45 (1), 159–169. 10.1002/hep.21493 17187424

[B57] Yuan J. Y. Wang H. J. Lin C. J. Liu D. W. Yu D. (2019). A novel GRU-RNN network model for dynamic path planning of mobile robot. Ieee Access 7, 15140–15151. 10.1109/access.2019.2894626

[B59] Zahednezhad F. Saadat M. Valizadeh H. Zakeri-Milani P. Baradaran B. (2019). Liposome and immune system interplay: challenges and potentials. J. Control. Release 305, 194–209. 10.1016/j.jconrel.2019.05.030 31121278

[B60] Zhang J. H. Li C. Yin Y. M. Zhang J. W. Grzegorzek M. (2023a). Applications of artificial neural networks in microorganism image analysis: a comprehensive review from conventional multilayer perceptron to popular convolutional neural network and potential visual transformer. Artif. Intell. Rev. 56 (2), 1013–1070. 10.1007/s10462-022-10192-7 35528112 PMC9066147

[B61] Zhang L. Tang Z. M. Zheng H. Zhong C. H. Zhang Q. (2023b). Comprehensive analysis of metabolome and transcriptome in fruits and roots of Kiwifruit. Int. J. Mol. Sci. 24 (2), 1299. 10.3390/ijms24021299 36674815 PMC9861564

[B62] Zhang Y. G. Pan G. F. Chen B. Han J. Y. Zhao Y. Zhang C. H. (2020). Short-term wind speed prediction model based on GA-ANN improved by VMD. Renew. Energy 156, 1373–1388. 10.1016/j.renene.2019.12.047

[B63] Zhang Y. L. Lei Y. Qi S. R. Fan M. X. Zheng S. Y. Huang Q. B. (2023c). Ultrasonic-microwave-assisted extraction for enhancing antioxidant activity of *Dictyophora indusiata* polysaccharides: the difference mechanisms between single and combined assisted extraction. Ultrason. Sonochemistry 95, 106356. 10.1016/j.ultsonch.2023.106356 PMC10014295 36905858

[B64] Zhong M. Y. Yan Y. Yuan H. S. Rong A. Xu G. Q. Cai F. J. (2022). Astragalus mongholicus polysaccharides ameliorate hepatic lipid accumulation and inflammation as well as modulate gut microbiota in NAFLD rats. Food and Funct. 13 (13), 7287–7301. 10.1039/d2fo01009g 35726797

[B65] Zhu W. J. Yu D. H. Zhao M. Lin M. G. Lu Q. Wang Q. W. (2013). Antiangiogenic triterpenes isolated from Chinese herbal medicine *Actinidia chinensis* planch. Anti-Cancer Agents Med. Chem. 13 (2), 195–198. 10.2174/1871520611313020002 22934692

[B66] Zou L. H. Cheng M. Hu K. L. Feng J. F. Tu L. X. (2024). Vesicular drug delivery systems for oral absorption enhancement. Chin. Chem. Lett. 35 (7), 109129. 10.1016/j.cclet.2023.109129

